# Fast Optical Sectioning for Widefield Fluorescence Mesoscopy with the Mesolens based on HiLo Microscopy

**DOI:** 10.1038/s41598-018-34516-2

**Published:** 2018-11-02

**Authors:** Jan Schniete, Aimee Franssen, John Dempster, Trevor J Bushell, William Bradshaw Amos, Gail McConnell

**Affiliations:** 1 Department of Physics, University of Strathclyde, Glasgow, G4 0NG United Kingdom; 2 Strathclyde Institute of Pharmacy and Biomedical Sciences, University of Strathclyde, Glasgow, G4 0RE United Kingdom; 3 MRC Laboratory of Molecular Biology, Cambridge Biomedical Campus, Cambridge, CB2 OQH United Kingdom

## Abstract

We present here a fast optical sectioning method for mesoscopy based on HiLo microscopy, which makes possible imaging of specimens of up to 4.4 mm × 3 mm × 3 mm in volume in under 17 hours (estimated for a z-stack comprising 1000 images excluding computation time) with subcellular resolution throughout. Widefield epifluorescence imaging is performed with the Mesolens using a high pixel-number camera capable of sensor-shifting to generate a 259.5 Megapixel image, and we have developed custom software to perform HiLo processing of the very large datasets. Using this method, we obtain comparable sectioning strength to confocal laser scanning microscopy (CLSM), with sections as thin as 6.8 ± 0.2 μm and raw acquisition speed of 1 minute per slice which is up to 30 times faster than CLSM on the full field of view (FOV) of the Mesolens of 4.4 mm with lateral resolution of 0.7 μm and axial resolution of 7 μm. We have applied this HiLo mesoscopy method to image fixed and fluorescently stained hippocampal neuronal specimens and a 5-day old zebrafish larva.

## Introduction

The Mesolens is a novel microscope objective lens that combines a high numerical aperture (NA) of 0.47 with a large field of view (FOV) of up to 6 mm. It has been used as the basis for CLSM to generate high quality optical sections of large specimens such as e12.5 mouse embryos with 700 nm lateral and 7 μm axial resolution^1^. The combination of large field of view and high spatial resolution means that confocal images of 20,000 pixels × 20,000 pixels are needed for Nyquist sampling. Even with a short pixel dwell time of 1 µs confocal imaging is slow, taking around 400 seconds per image. Z-stacks of large volume specimens can take from several hours up to days or even weeks to acquire, depending on the total volume, number of channels used, and any frame averaging to improve the signal-to-noise ratio (SNR). Although the Mesolens has proven utility in biomedicine^1,2^, a fast widefield optical sectioning method is essential to reduce acquisition time to make the Mesolens suitable for rapid high-resolution imaging of large volume specimens.

Widefield techniques capable of performing optical sectioning are highly sought after in biological imaging to reduce photodamage and photobleaching as well as increasing contrast (reduction of out-of-focus blur) at high acquisition speed. Each method has its own strengths and weaknesses and the choice depends on the specimen of interest.

Widefield two-photon microscopy (W2PM) has been reported for *in vivo* imaging^3^ and it has been shown to produce less photobleaching than single-photon excitation without the need to scan the beam over the field of view, capable of reaching 100 Hz acquisition speed^4^. Furthermore, W2PM can perform optical sectioning when the peak intensity overcomes the threshold for 2P absorption only in the focal plane using temporal focusing^5,6^. On the downside, the 2P absorption process is non-linear and requires ultra-short laser pulses on the order of few hundred femtoseconds. W2PM would require very high peak intensity radiation propagating through the Mesolens to overcome the threshold to excite fluorescent molecules at the sample, potentially damaging the optical elements.

Selective plane illumination microscopy (SPIM), also called light sheet microscopy (LSM) has received a lot of attention in recent years. Since its original conception in 1902^7^, SPIM has been reintroduced for modern microscopy techniques^8^. The biggest advantage is the lack of illumination outside the focal plane. This leads to minimal phototoxicity and photobleaching. However, the side-on illumination can lead to inhomogeneous brightness across the field of view and scattering samples can cast ‘shadows’ in the lateral direction. Techniques have evolved to counter these effects, e.g. dual side illumination^9^ and the use of non-diffracting beams allow the creation of thin light sheets over a comparably large FOV up to 1 mm^10^. A tenfold increase in light sheet FOV coverage over standard Gaussian light sheets has been reported^11^, but the total volume is still ~10 times less than can be accommodated with the Mesolens at similar spatial resolution (estimated based on a CFI S Fluor 10x objective lens with 0.5 NA and 1.2 mm working distance). For the Mesolens, because of its unique combination of high NA and large FOV, SPIM is not capable of generating a light sheet that has a long enough Rayleigh length to cover the full field of view at ~7 µm thickness. The only method to cover such a large FOV with a thin sheet would be to scan the light sheet and acquire multiple images or use a rolling shutter as presented in recent work^12–14^. This approach would be similar to stitching and tiling on standard microscope lenses but with the FOV remaining unchanged and the illumination moving through several positions in the FOV. Stitching and tiling methods would defeat the purpose of using the Mesolens and greatly increase acquisition time. The camera currently in use on the Mesolens does not provide a rolling shutter function and no camera with a rolling shutter is commercially available that provides high enough resolution to accommodate the Mesolens.

Structured illumination microscopy (SIM) is a super-resolution optical imaging technique that inherently provides optical sectioning as it only regards modulated, in-focus signal for the final image^15,16^. A related method called HiLo microscopy (“Hi” and “Lo” representing the high and low spatial frequency components, not to be confused with HILO, highly inclined and laminated optical sheet^17^) has been developed recently^18^ which makes use of the optical sectioning capability of SIM without any super-resolution content. Only two images are required for HiLo microscopy, which can reduce phototoxicity and photobleaching effects^19,20^ as opposed to three images for optical sectioning SIM^21^ or at least 4 images for 2D super-resolution SIM techniques^19^. HiLo uses post processing of one structured and one uniform illumination image to achieve the result. The optical sectioning strength of HiLo has been reported to be comparable to CLSM and acquisition speed is in principle only limited by the camera exposure time^22^. The HiLo method can be implemented inexpensively by using a diffuser to create laser speckle with coherent (laser) light or with a grating and incoherent light. This diffuser-based method of generating laser speckle has been reported to be more robust than using a grating to study scattering samples^22^ and the contrast of speckle can be easily adjusted to suit different samples^23^. SIM does provide super-resolution, but it is not straightforward to implement with the Mesolens: structured illumination would have to be introduced in the back aperture plane of the condenser because the back focal plane of the objective is not accessible in the current Mesolens design. As a direct result, thick samples could not be imaged because the grating contrast would quickly deteriorate due to scattering.

We therefore chose to implement HiLo microscopy in transmission illumination with laser speckle illumination with the Mesolens to obtain optical sections and investigated sectioning strength, quality and speed compared to CLSM. We elected to write our own script to perform the computational side of HiLo microscopy in MATLAB and compared its performance to an ImageJ plugin which was written by the developers of HiLo microscopy.

## Results

The HiLo method uses two images, one with uniform illumination and one with laser speckle illumination to obtain optical sections by evaluating the local contrast in the imaged speckle and several stages of filtering. A single parameter, σ, that governs the filter frequency in Fourier space was used to adjust the optical section thickness. The process is detailed in the Materials and Methods section and published work^18,22,23^.

**Figure 1:**
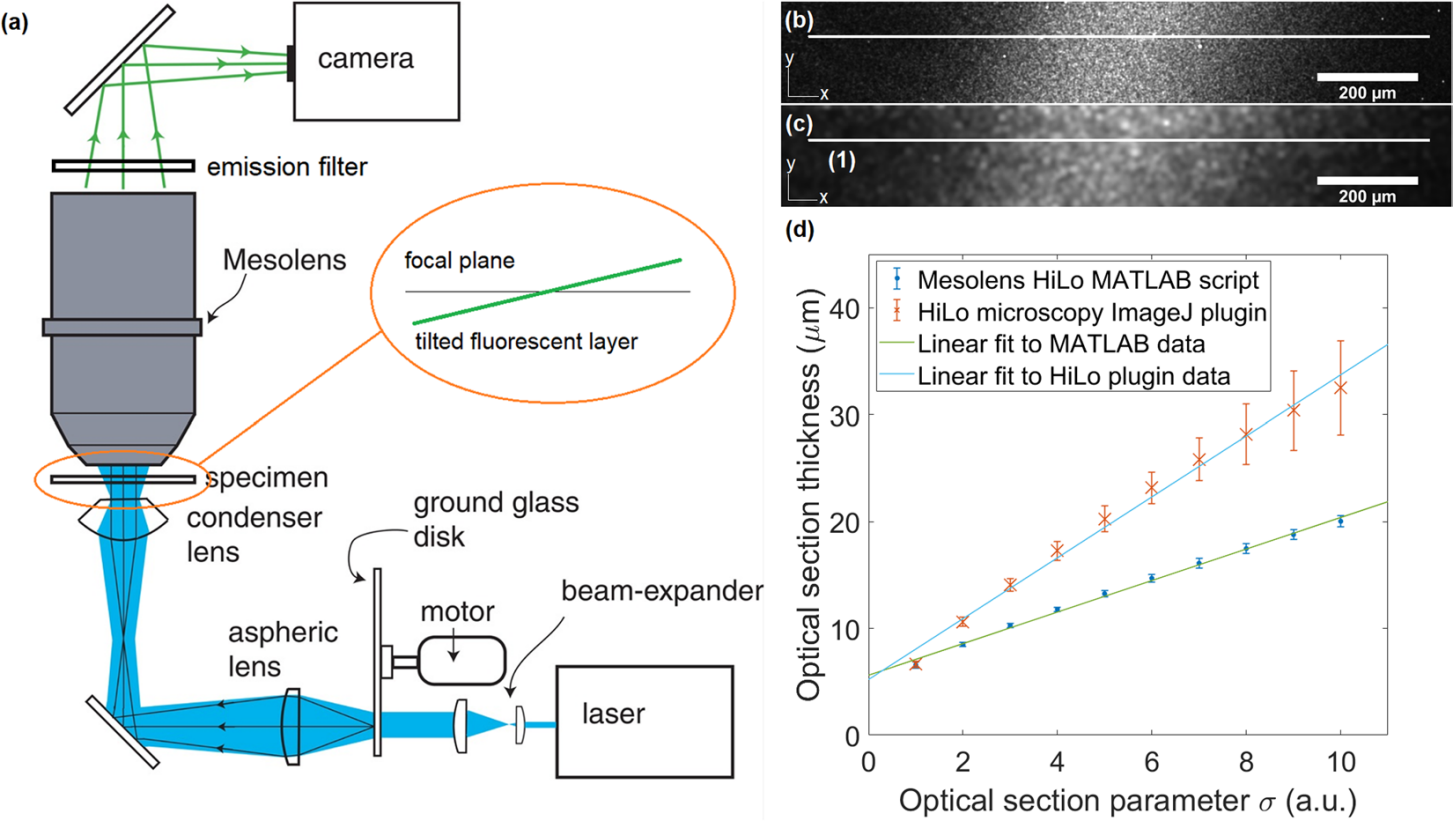
Comparison of optical sectioning strength. A tilted layer of fluorescent dye was processed with the HiLo ImageJ plugin and our MATLAB script. (**a**) Shows the experimental setup depicting the fluorescent layer tilt in the inset. The tilt is exaggerated to make clear that only a narrow strip in the centre was in focus. An example of the HiLo processed layer is shown for (**b**) the ImageJ plugin and (**c**) the MATLAB script with the optical sectioning parameter σ set to 1 for both processing modalities which corresponded to approximately the same optical section thickness. The thickness as a function of σ (**d**) was obtained by processing the same data of the tilted fluorescent layer with the plugin and our MATLAB script and calculating the average full-width-half-maximum (FWHM) of Gaussian fits to five horizontal intensity line plots through the processed images. (1) Shows an example of such a horizontal line plot, the line is made thicker to improve visualisation but was only one pixel thick for the measurement. The two processing modalities reached a minimum section thickness of 6.6 ± 0.3 μm (plugin) and 6.8 ± 0.2 μm (MATLAB) at the lowest setting for σ. The graininess is an example of artefacts that can arise when speckle structure translated through to the final image. This could be avoided in thicker samples or by setting σ to higher values, i.e. thicker optical sections.

**Figure 2:**
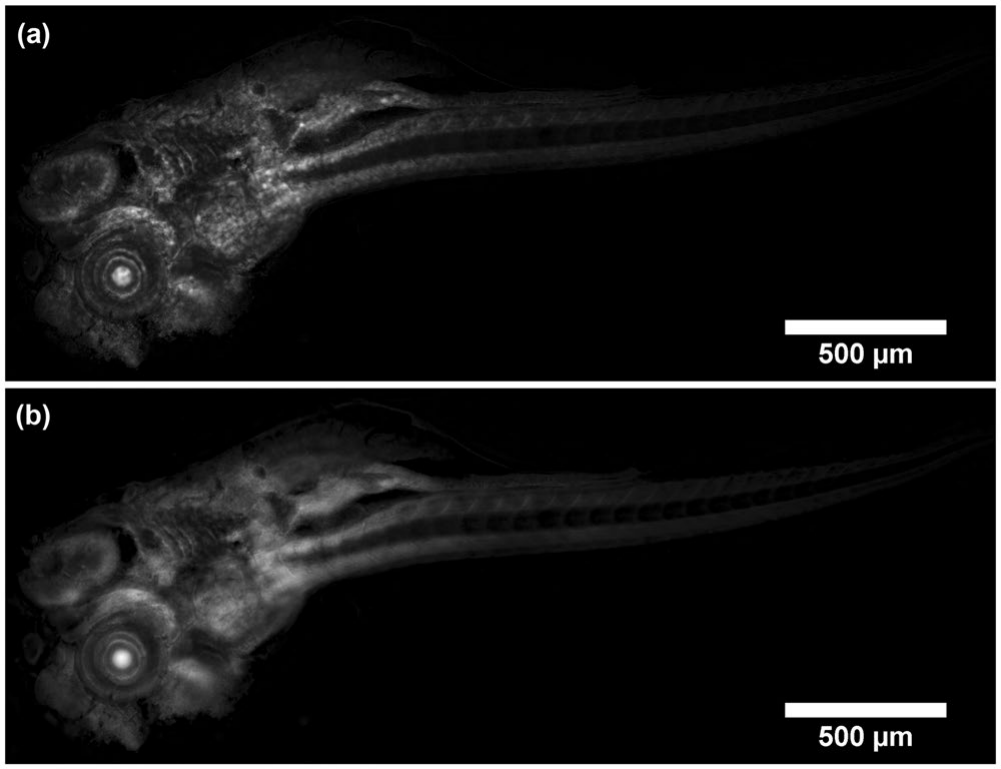
Example of artefact formation in HiLo images. (**a**) Shows artefacts which can appear if the contrast evaluation window is chosen to be too small to include enough imaged speckle grains. In this scenario, the final image contains structure of the speckle illumination. (**b**) Shows a HiLo processed image which has proper contrast evaluation window size and does not contain the illumination structure.

It was found that the lowest setting for the optical sectioning parameter σ where the dependence of optical section thickness on σ was still linear corresponded to a frequency of $\frac{1}{10}{{\rm{pixel}}}^{-1}$. The scaling in our MATLAB script was therefore adjusted to have this value as a minimum when σ was set to 1. At this setting an optical section thickness of 6.8 ± 0.2 µm (mean ± standard deviation of five measurements) was measured by evaluating the average FWHM of Gaussian fits to horizontal intensity line plots through the processed image of a tilted fluorescent layer discussed in the Materials and Methods section. Similarly, the minimum optical section thickness of the ImageJ plugin attainable was measured at 6.6 ± 0.3 µm. Figure 1 shows the tilted layer processed with our MATLAB script compared to the HiLo ImageJ plugin and optical section thickness measurements at σ settings ranging from 1 to 10 for the plugin and MATLAB script respectively. The images of the tilted layer also showed an example of artefact formation that was particularly common in thin samples; speckle pattern structure was visible in the final image because the contrast evaluation and thus the weighting function followed the intensity distribution of the imaged speckle structure. This effect disappeared when σ became larger or the sample was thicker. Figure 2 shows an example of this artefact formation in the 5-day-old zebrafish specimen compared to an artefact-free image of the same focal plane.

As expected from other work in the microscopic domain^23^, it was found that the speckle pattern for the structured illumination image needed to be coarser when imaging thicker samples (more than ~100 μm thick) but also depended on how densely labelled the sample was. In the ideal case, the transverse size of an imaged speckle grain was determined by the illumination NA^18,22^. However, for thicker samples the imaged grain size was increased to approximately 20 pixels (~5 μm at 9x chip shifting) to maintain high contrast in the difference image for the in-focus regions of the image. The choice of the σ parameter had to be made according to the coarseness of said pattern such that several imaged grains would fit in the sampling window for contrast evaluation to avoid the above-mentioned artefacts in the final image. Specimens of fixed and fluorescently stained hippocampal mouse neurons and a 5-day-old zebrafish larva specimen were imaged with the Mesolens and processed in MATLAB using suitable σ settings to evaluate performance of the method with thick and uncleared biological specimens.

**Figure 3:**
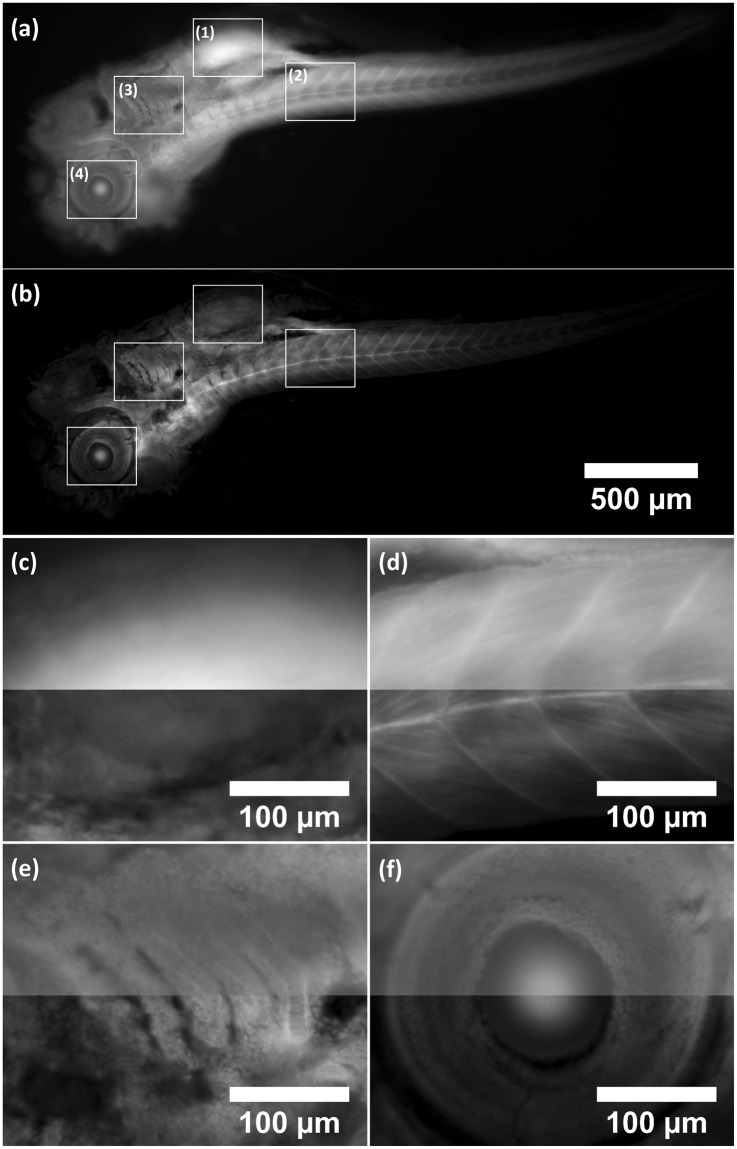
Standard widefield image (**a**) compared to Mesolens HiLo-processed optical section (**b**) of zebrafish stained with Acridine Orange. Insets (**c**–**f**) show zoomed-in regions of interest (1–4) where the widefield and HiLo image were merged together (top half was widefield, bottom half was HiLo image). Contrast improvement was evident across the whole image and regions of interest showed fine detail that was barely noticeable in the widefield image. Optical sectioning parameter σ was set to 2, corresponding to 8.7 ± 0.1 μm. With σ set to 1 there were too many artefacts. There was still a hint of inhomogeneous brightness with σ = 2 but not so severe that false detail emerged in the final image. Setting σ higher would have resulted in an unnecessarily thick section.

The ~150 μm thick zebrafish stained with Acridine Orange was imaged and HiLo processed in MATLAB with σ = 2. This was the lowest value for the combination of sample thickness and choice of speckle coarseness where no artefacts were observed in the final image. The excitation wavelength was 488 nm with ~4 mW laser power at the sample. The fluorescence signal was filtered at 540 nm with spectral full-width-half-maximum of ~20 nm and exposure time of the camera detector was 400 ms for each of the nine sensor positions. Supplementary video 1 shows the data as a z-series video, with the z-stack comprising 61 images taken with 3 μm steps in axial position. The FOV was cropped to roughly the size of the zebrafish (3.2 mm × 1.2 mm). The zoomed-in eye showed improved contrast. Individual cells were clearly visible as well as the macroscopic structure of the surrounding tissue. Figure 3 shows a transmission illumination widefield image (the uniform image also used for processing) compared to an optical section from the 61-image z-stack at approximately 100 μm depth in the sample. An improvement in contrast was evident across the whole field of view and zoomed-in regions of interest revealed fine detail that was not clearly visible in the widefield image.

**Figure 4:**
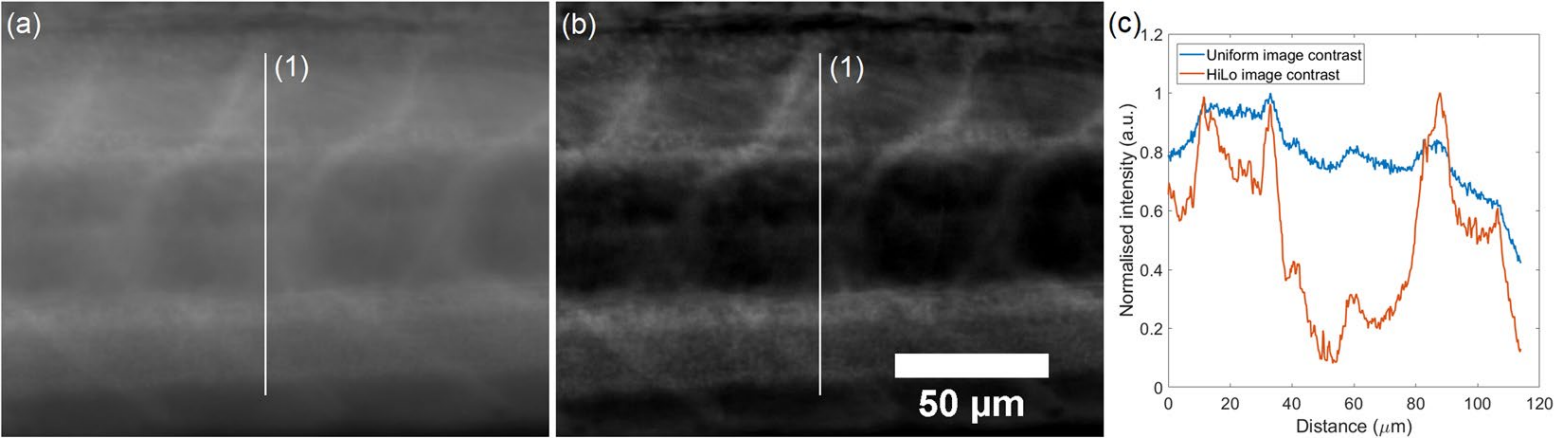
Contrast improvement of Mesolens HiLo over widefield. (**a**) is a widefield image, (**b**) the HiLo processed image and (**c**) are the respective plots of vertical line (1). The images are of the same region of the spine as in Fig. 2 but at a different z-position. Z-position was chosen to specifically highlight the contrast improvement. Contrast was calculated by dividing peak intensity value by the minimum value (out-of-focus background). Results were 2 for the widefield image and 12 for the HiLo processed image, i.e. a six-fold increase in contrast. Scale bar applies to both images.

To quantify the contrast increase, the fluorescence intensity was measured from the peak value and background. Figure 4 shows the spine section of the same zebrafish as in Fig. 3 in grayscale. The peak fluorescence signal intensity of the vertical line plot was measured and divided through by the minimum value (out-of-focus background). Contrast in the widefield image was 2 and contrast in the HiLo image was 12, i.e. a six-fold increase in contrast in this example.

**Figure 5:**
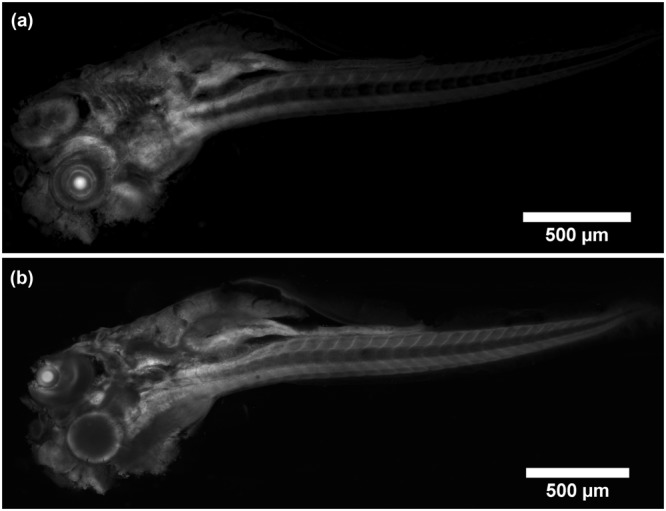
Comparison between HiLo processed optical section (**a**) and confocal laser scanning microscopy optical section (**b**). The two sections are approximately in the same focal plane of the same zebrafish which was used for Figs 2–4. However, the confocal data was obtained in epi-illumination while the HiLo data was taken with transmission illumination. The difference in illumination accounts for the major discrepancies in the observed structure. Furthermore, the confocal data had more even intensity across the field of view.

Figure 5 shows a direct comparison between HiLo and laser scanning confocal microscopy on the Mesolens. The confocal data had even intensity across the whole image regardless of sample thickness whereas the HiLo processed image was significantly brighter in areas where the specimen was very thick (e.g. the brain) and dim where the specimen was thin (e.g. the spine). The structure of the zebrafish in the confocal data differed from the HiLo processed zebrafish data due to different illumination techniques (epi-illumination for confocal and transmission illumination for HiLo). Supplementary video 2 shows the complete dataset of the 47-image z-stack of the CLSM data. The data were down-sampled to reduce file size for presentation.

For thick, turbid specimens such as the 150 um thick zebrafish, the in-focus speckle contrast deteriorated due to out-of-focus blur such that the weighting function was underestimated in regions where the specimen was very thick compared to the edges, despite both regions were in focus. This effect led to inhomogeneous brightness of the final image but was opposite to the effect mentioned previously where different areas were weighted equally but had different intensity to begin with. Supplementary video 4 shows an Eosin stained *Daphnia* sample that was 300 µm at its thickest region where this effect was obvious.

For sparse, comparatively thin samples such as a neuronal cell monolayer, the sectioning parameter σ was set to 1. This was the lowest setting at which the sectioning curve in Fig. 1 behaved linearly and was chosen to obtain the thinnest sections without speckle artefacts in the final image. Supplementary video 2 shows fluorescently stained mouse hippocampal neurons sectioned at this setting. The excitation wavelength, detection bandpass and average power at the sample were the same as for the zebrafish specimen. The camera exposure setting was 200 ms (for each sensor position). Optical sectioning revealed the axial extent of dendrites and cell bodies (soma) and improved contrast to clearly show the unstained centre of the soma where the nucleus is located. Acquiring the data took ~27 minutes per stack (uniform and speckle illumination) and another ~26 minutes to process in MATLAB.

Acquisition of one image pair (speckle and uniform illumination) took 1 minute for the full 4.4 mm FOV and Nyquist sampled images (9x chip-shifting). It was found that the frame time, i.e. the time it took to acquire an image and start the next acquisition, was mainly taken up by transferring the image from the camera to the PC and saving it on the hard drive. Hence the actual exposure did not significantly impact the time to acquire a whole z-stack. The 25-image z-stack of neurons took ~27 minutes to acquire per illumination at 200 ms camera exposure. Processing a full FOV full resolution image-stack in MATLAB took 1 minute per image pair. Image processing was performed post acquisition and did not increase imaging time. Furthermore, the same raw images could be processed at different settings without the need to re-acquire data. Compared to CLSM this method acquires raw data 30 times faster, including processing it is still 15 times faster, estimated based on a Nyquist sampled CLSM full FOV image with three times averaging (30 minutes acquisition time per image).

## Discussion

We have shown here a fast widefield optical sectioning method for the Mesolens using HiLo microscopy capable of section thickness of 6.8 ± 0.2 µm over the full FOV of 4.4 mm, on par with CLSM on the Mesolens which can generate sections of 5 μm thickness by imaging a thin fluorescent layer which was set at a tilt. Imaging fluorescent beads was originally considered to determine optical section thickness, however, the HiLo process collapses to a simple high pass filter and would not represent the optical sectioning capability which relies on contrast evaluation over a window containing several imaged diffraction limited speckle grains. That is the reason the tilted fluorescent layer was chosen as a measure of optical sectioning thickness. The lateral resolution remained unaffected by HiLo microscopy because the high spatial frequency, i.e. high resolution, content of the final image was obtained by high-pass filtering the uniform image. Since the uniform image was a standard widefield camera image with diffraction limited resolution, no fine detail was lost^23^. This method presents a significant speed advantage over CLSM at comparable sectioning strength, being 30 times faster in raw data acquisition. However, CLSM did achieve even intensity regardless of specimen thickness whereas HiLo images were brighter in areas where the specimen was thick and dimmer where the specimen was thin. This effect was a direct consequence of how optical sectioning is achieved in either method; CLSM optically rejects out-of-focus background by the confocal pinhole aperture in the detection beam path. HiLo rejects out-of-focus background computationally, but each pixel contains in-focus and out-of-focus intensity to begin with. Thus, a pixel in a thick area of the specimen will be brighter in the final image than a pixel in a thin area of the specimen if both are considered equally in-focus by the weighting function. Contrary to this effect, high out-of-focus background decreased the speckle contrast in thick samples which lead to underestimation of the weight function. Direct comparison with CLSM was also difficult due to the difference in illumination. CLSM was performed in epi-illumination whereas HiLo mesoscopy was performed in transmission illumination which lead to significant discrepancies in the images.

We elected to write our own script in MATLAB rather than use the existing ImageJ HiLo plugin. Although the plugin performed very well when we initially tested it with small datasets, it was very slow (~16 times slower than the current MATLAB implementation) when processing large Mesolens data and in some cases, data could not be processed at all due to memory limitations (even when using a server for processing). Using a homebuilt script further allowed us to change parameters like optical sectioning factor, low frequency scaling factor, contrast evaluation window size and filter frequencies more freely while knowing exactly what impact each had on the final image. The main speed limitation of widefield acquisition on the Mesolens was not the camera exposure but rather handling of the data itself. At full FOV, camera images are approximately 500 Mb large. Each image needed to be transferred from the camera buffer to the PC’s hard drive before a new image could be acquired. As a direct result, reducing the camera exposure time by 80% (1000 ms to 200 ms) only reduced acquisition time for a 25-image z-stack from 32 minutes to 27 minutes (~15% time reduction). The chip shifting mechanism of the VPN-29MC was necessary to obtain Nyquist sampled images but shifting the detector chip through nine positions meant that exposure time for one frame was 1800 ms (9 times 200 ms) at minimum. The fastest practical exposure setting was 200 ms, as below this exposure time the readout of the CCD detector array increased frame time to 200 ms regardless (according to VPN-29MC user manual). A single detector array of sufficiently small pixel size would potentially increase acquisition time but would not get around the data handling constraints. Furthermore, no detector with such high pixel number and sensor size compatible with the Mesolens is commercially available at present and custom-built detectors would come at very high cost.

Despite these drawbacks, HiLo mesoscopy is an excellent alternative to CLSM for the Mesolens and offers some advantage over SPIM. Unlike SPIM, HiLo obtains uniform section thickness over the full FOV without the need to stitch and tile or use a rolling shutter comparable in sectioning strength to CLSM. The setup for HiLo is considerably simpler with just a diffuser in an otherwise ordinary transmission illumination setup. SPIM would require side illumination, potentially casting lateral shadows across the FOV at optical section thickness upwards of 20 µm for standard Gaussian beams. SPIM also requires specialised sample chambers whereas HiLo can operate with the same sample preparation procedures already in place for the Mesolens’ other imaging modalities.

It has been discussed in detail^23^ that the strong out-of-focus background in thick samples presents a problem for HiLo imaging by reducing in-focus speckle contrast and hence making it more difficult to distinguish in-focus from out-of-focus regions in the image. To maintain high speckle contrast, a coarser speckle illumination pattern was used for thick, densely labelled samples. Rather than decreasing the illumination NA, we chose to implement a variable beam expander in the illumination beam path. By changing the beam diameter illuminating the diffuser, we could adjust the speckle coarseness to suit the sample and maintain sectioning capability, albeit with thicker sections. Although the sections were thicker than the axial resolution, approximately 10 μm for σ = 3, sectioning was still superior to what SPIM is capable of on this FOV. The above-mentioned contrast issue affects axial resolution only since lateral resolution is determined by the microscope system.

## Methods

### Theoretical background of Hilo microscopy

HiLo microscopy was described in detail by its developers Lim and Mertz elsewhere^18,22^. The core equation that summarises the optical sectioning capability is

1 $${C}_{\delta s}^{2}({\rm{z}})={A}_{s}{\int }^{}{|BP(k)|}^{2}{|OT{F}_{det}(k,z)|}^{2}OT{F}_{ill}(k,0){d}^{2}k,$$

where ${C}_{\delta s}({\rm{z}})$ is the imaged speckle contrast, *A*_*s*_ is the average transverse area of a speckle grain, *BP*(*k*) is the Bandpass filter, *OTF*_*det*_(*k, z*) is the detection optical transfer function and *OTF*_*ill*_(*k*, 0) is the illumination optical transfer function. Equation (1) has been repeated from^22^ and the interested reader can find the full derivation there.

We repeat only the basic principles of the HiLo process here which are necessary to reproduce our results. HiLo microscopy performs optical sectioning of fluorescent samples by segmenting the image using contrast evaluation of the difference image of a structured illumination image and a uniform illumination image and obtaining a weighting function as a result. The uniform image i_u_ is a simple widefield fluorescent image. To obtain the structured illumination image i_s_, the sample is illuminated by a random laser speckle pattern. The in-focus high spatial frequencies of the image are obtained by simply applying a gaussian high-pass filter to a Fourier transformed uniform image such that:

2 $${{\rm{i}}}_{{\rm{HP}}}={ {\mathcal F} }^{-1}({ {\mathcal I} }_{{\rm{u}}}\times {\rm{HP}}),$$

where i_HP_ is the high-pass filtered uniform image, ${ {\mathcal F} }^{-1}$ is the inverse Fourier Transform, ${ {\mathcal I} }_{{\rm{u}}}$ is the Fourier Transform of i_u_ and HP is a gaussian high-pass filter with cut-off frequency k_c_, such that HP(k_c_) = 1/2.

The high spatial frequencies are inherently in focus and thus do not need to be further processed. To obtain the in-focus low spatial frequencies, first the difference image, i_d_, must be calculated

3 $${{\rm{i}}}_{{\rm{d}}}={{\rm{i}}}_{{\rm{s}}}-{{\rm{i}}}_{{\rm{u}}}.$$

Subtracting i_u_, the uniform illumination image, from i_s_, the speckle illumination image, removes the sample induced bias and allows the evaluation of local speckle contrast to be performed on the variations of the speckle pattern only.

The local contrast of speckle grains tends to zero with defocus and thus allows to distinguish between in-focus and out-of-focus signal. This decay to zero can be accelerated by applying a bandpass filter to i_d_ prior to contrast evaluation

4 $${\rm{BP}}=\exp (-\frac{{{\rm{k}}}^{2}}{4{{\rm{\sigma }}}^{2}})-\exp (-\frac{{{\rm{k}}}^{2}}{2{{\rm{\sigma }}}^{2}}),$$

where BP is the bandpass filter, generated by subtracting two Gaussian lowpass filters, k is the spatial frequency and σ is the bandpass filter standard deviation.

Correct evaluation of local speckle contrast is key to separate in-focus from out-of-focus signal. Local contrast evaluation can be performed by calculating the quotient of standard deviation and mean in a local neighbourhood with a sliding window^23,24^.

5 $${{\rm{C}}}_{\langle {\rm{\Lambda }}\rangle }=\frac{{{\rm{sd}}}_{\langle {\rm{\Lambda }}\rangle }}{{\mu }_{\langle {\rm{\Lambda }}\rangle }}.$$

where C_〈Λ〉_ is the contrast in the local neighbourhood, evaluated within a sliding window of side length Λ (pixels). sd_〈Λ〉_ and µ_〈Λ〉_ are the standard deviation and mean intensity in the local neighbourhood respectively.

The side length Λ of the sliding window is determined depending on the cut-off frequency k_c_ as described in reference^23^.

6 $${\rm{\Lambda }}=\frac{1}{2{{\rm{k}}}_{{\rm{c}}}}$$

Applying the local contrast as a weighting function to i_u_ results in a coarse image of in-focus low spatial frequencies.

7 $${{\rm{i}}}_{{\rm{su}}}={{\rm{i}}}_{{\rm{u}}}\times {{\rm{C}}}_{\langle {\rm{\Lambda }}\rangle },$$

where i_su_ is the weighted uniform illumination image. By applying a gaussian low-pass filter LP complementary to HP, i.e. LP + HP = 1, the in-focus low spatial frequencies are obtained

8 $${{\rm{i}}}_{{\rm{LP}}}={ {\mathcal F} }^{-1}({{\mathscr{ {\mathcal I} }}}_{{\rm{su}}}\times {\rm{LP}}),$$

where i_LP_ is the in-focus low spatial frequency image, ${ {\mathcal I} }_{{\rm{su}}}$ is the Fourier Transform of i_su_ and LP is the complementary low-pass filter. To ensure a smooth transition between i_LP_ and i_HP_ , a scaling factor is calculated

9 $${\rm{\eta }}={ {\mathcal I} }_{{\rm{HP}}}({{\rm{k}}}_{{\rm{c}}})/{ {\mathcal I} }_{{\rm{LP}}}({{\rm{k}}}_{{\rm{c}}}),$$

where η is the scaling factor, ${ {\mathcal I} }_{{\rm{HP}}}({{\rm{k}}}_{{\rm{c}}})$ and ${ {\mathcal I} }_{{\rm{LP}}}({{\rm{k}}}_{{\rm{c}}})$ are the Fourier Transforms of i_HP_ and i_LP_ respectively evaluated at the cut-off frequency k_c_.

The final optically sectioned HiLo image is obtained by adding the in-focus high and low spatial frequency images together.

10 $${{\rm{i}}}_{{\rm{HiLo}}}={{\rm{i}}}_{{\rm{HP}}}+{\rm{\eta }}\ast {{\rm{i}}}_{{\rm{LP}}}$$

Where i_HiLo_ is the final optically sectioned image. By setting k_c_ = 0.18σ^22,23^, the optical sectioning strength can be controlled by changing only the σ parameter.

### Experimental setup

A schematic of the setup is shown in Fig. 1a. A Coherent Sapphire 488-10 CDRH laser was used as a light source that emitted light at 488 nm at 4 mW peak power at the sample. The beam was guided through a variable beam expander (Thorlabs BE02-05, 2x-5x variable zoom Galilean beam expander), increasing the beam diameter from 1 mm to a minimum of 2 mm and maximum of 5 mm. The final beam diameter resulted in coarse speckle (beam expander set 2x) with higher contrast in thick specimen at the cost of optical sectioning strength or fine speckle (beam expander set to 5x) allowing thin sectioning but contrast degradation in thick samples as described in the Results section. Subsequently the beam illuminated a 1500 grit ground glass diffuser (DG20-1500, Thorlabs). The diffuser was glued to a DC motor controlled via an Arduino Uno board connected to a PC via USB. It was imaged onto the back aperture of the 0.6 NA Mesolens condenser (Mesolens Ltd.) using an aspheric lens with 0.6 NA (ACL5040U-A, Thorlabs). With the diffuser stationary, a speckle pattern was generated in the sample. Rotating the diffuser via the DC motor (6/9 V, 12000 ± 15% rpm) resulted in uniform illumination, thus allowing acquisition of both uniform and speckle illumination images in quick succession at one minute raw acquisition time per image pair on the full 4.4 mm FOV of the camera. The samples were imaged by the Mesolens onto a camera detector. The triple band emission filter was part of the commercial Mesolens system and transmitted light at 470 ± 10 nm, 540 ± 10 nm and 645 ± 50 nm. Not shown in this diagram in Fig. 1a are two mirrors that are placed before and after the beam expander to guide the laser beam. Images were acquired with a thermoelectric Peltier cooled camera (VNP-29MC, Vieworks) with a chip-shifting mechanism. The chip-shifting mechanism was essential to benefit from the large FOV and high resolution (700 nm lateral, 7 µm axial^1^) provided by the Mesolens. The camera port on the Mesolens system contains a focusing lens providing an additional magnification of 2x bringing the total system magnification to 8x. The technical specifications of the Mesolens system have been published elsewhere^1^. The camera could be operated without chip-shift at a resolution of 6576 × 4384 pixels (28.8 Megapixel), with 4x chip-shift at 13152 × 8768 pixels (115.3 Megapixel) and with 9x chip-shift at 19728 × 13152 pixels (259.5 Megapixel). For HiLo imaging with the Mesolens, the chosen mode was always 9x chip-shift. In this mode, the sampling rate was 4.46 px/µm, corresponding to a 224 nm pixel size, satisfying Nyquist sampling. The sampling rate of the image was determined by imaging a 1 mm graticule (Graticule Ltd., Tonbridge, England) and equating the known distance in µm to a distance in pixels in ImageJ. The minimum frame time of the Vieworks camera was 200 ms resulting in acquisition time for one full FOV image with 9x pixel shift of 1800 ms excluding time to transfer the image data from the camera to the PC which usually took on the order of 10 seconds. In practice, acquisition of one image took 12–15 seconds including transfer of data and beginning of new image capture.

### Data processing

To process the speckle and uniform images a MATLAB (R2016b version 9.1.0.441655, 64 bit, MathWorks, Inc.) script^25^ was written that performed HiLo imaging in the same manner as described in the previous section. This allowed more control over individual parameters (optical sectioning factor, low frequency scaling and cut-off frequency) and opened the possibility to use the parallel processing toolbox of MATLAB to use a graphics processing unit (GPU). Because of the file size of Mesolens images, it was necessary to process z-stacks of samples on a server as commercially available desktop PCs do not have enough memory to open or process such large files. The server was a Dell PowerEdge R740 with 1TB RAM and an NVIDIA Quadro P4000 GPU with 8GB video memory.

### Measuring the optical sectioning capability of Mesolens HiLo

To determine the optical sectioning strength of the HiLo method, a thin fluorescent layer was set at a tilt by wedging a microscope slide under one end of the sample microscope slide, a known height difference between the two ends of the sample slide was introduced. The resulting image then showed the fluorescent layer as a narrow strip as shown in Fig. 1, coming into focus in the centre of the field of view and go out of focus towards the left and right. Since the length of the sample slide is also known, a measured FWHM in the lateral direction can be translated into an axial FWHM, thus giving an experimental measure of the optical section thickness. This method was adapted after^26^. To prepare the thin fluorescent layer, first a 170 μm thick microscope cover slip (22 mm × 22 mm, #1.5, Thermo Fisher Scientific) was rinsed in dry acetone (Acetone 20066.330, VWR Chemicals). It was then submerged in an APTMS-acetone (3-Aminopropyldrimethoxysaline, 281778-100 ML, Sigma Aldrich) solution for six hours (0.2 mL APTMS, 9.8 mL of dry acetone). After this period, the cover slip was rinsed three times in dry acetone and blow-dried with compressed air. The cover slip was put in a 10 μM solution of fluorescein salt (Fluorescein sodium salt, 46960-25G-F, Sigma Aldrich) in distilled water. Care was taken to only let one side of the cover slip get in contact with the fluorescein solution to avoid having two thin fluorescent layers (one on either side). The bath was carefully wrapped in aluminium foil and left overnight in a dark place. The next day, the cover slip was rinsed with distilled water twice and again blow-dried with compressed air. Finally, the cover slip was mounted on a microscope slide with the dye-coated surface in contact with the slide and was sealed with nail varnish. Imaging was done with glycerol immersion.

### Mouse hippocampal neuron sample preparation

The mouse hippocampal neuron sample was prepared from C57BL/6J pups (1-2 days old) as described previously^27,28^ and fluorescently stained^29,30^. All experimental procedures were performed in accordance with UK legislation including the Animals (Scientific Procedures) Act 1986 and with approval of the University of Strathclyde Animal Welfare and Ethical Review Body (AWERB). In short, neurons were fixed in ice-cold 4% paraformaldehyde (PFA). The sample was then incubated with a primary anti-mouse antibody (anti-βIII-tubulin (1:500), Sigma-Aldrich) and fluorescently labelled using a secondary antibody (anti-rabbit AlexaFluor 488 (1:200), Thermo Fisher Scientific). The fixed and stained sample was mounted onto a glass microscope slide (VWR, UK) using Vectashield mounting medium (H-1200, Vector Laboratories) and imaging was performed with glycerol immersion on the Mesolens.

### 5-day-old zebrafish larva sample preparation

The zebrafish were fixed in ethanol: glacial acetic acid at a 3:1 ratio at 4 °C for 72 hours, then washed in 100% ethanol and rehydrated progressively in ethanol/saline solutions before staining in 0.01% acridine orange (A1301, Thermo Fisher Scientific) in phosphate-buffered saline with gentle agitation before dehydration in an ethanol/ saline series. The dehydrated specimens were washed three times in absolute ethanol (dried with molecular sieve) and then transferred via xylene, changed twice and left in xylene for two hours and checked for transparency. They were tumbled gently overnight in a solution Fluoromount^31^ (Fluoromount is no longer available commercially: we would advise Histomount (Thermo Fisher Scientific) as similar substitute) before mounting in a single-cavity slide under a standard coverslip, with the specimen left uncovered to facilitate the evaporation of the xylene solvent and more mountant being added to reduce shrinkage. Imaging was performed with glycerol immersion on the Mesolens and custom built acquisition software based on WinFluor^32^.

## Electronic supplementary material

Supplementary video captions

Supplementary video 1

Supplementary video 2

Supplementary video 3

Supplementary video 4

## Data Availability

The datasets generated during and/or analysed during the current study are available from the corresponding author on reasonable request.
