# Supplementary material for: Fast Optical Sectioning for Widefield Fluorescence Mesoscopy with the Mesolens based on HiLo Microscopy
Source: Sci Rep. 2018 Nov 2;8:16259. doi: 10.1038/s41598-018-34516-2 (PMC6215018; doi:10.1038/s41598-018-34516-2)
Supplement: Supplementary file 1 — Supplementary video captions [file 41598_2018_34516_MOESM1_ESM.docx]

Fast Optical Sectioning for Widefield Fluorescence Mesoscopy with the Mesolens based on HiLo Microscopy

Jan Schniete^1^*, Aimee Franssen^2^, John Dempster^2^, Trevor Bushell^2^, William Bradshaw Amos^1,3^, Gail McConnell^1^

^1^ Department of Physics, University of Strathclyde, Glasgow, G4 0NG, United Kingdom

^2^ Strathclyde Institute of Pharmacy and Biomedical Sciences, University of Strathclyde, Glasgow, G4 0RE, United Kingdom

^3^ MRC Laboratory of Molecular Biology, Cambridge Biomedical Campus, Cambridge, CB2 OQH, United Kingdom

* jan.schniete@strath.ac.uk

# Supplementary Data information (captions)

**Supplementary video 1: Acridine Orange stained zebrafish.** The first part of the movie shows a cropped FOV (smaller than the Mesolens full FOV but large enough to see the whole zebrafish) 61-image z-series (at 3 μm z-steps) of a zebrafish. The second part of the movie shows a software zoom to the region where the eye is located. The third part is, again, a z-series of the ROI, individual cells are clearly visible, and the macroscopic structure of the sample becomes obvious. The sample was excited at a wavelength of 488 nm and fluorescence was detected at 540 nm as before. The optical sectioning parameter σ was set to 3 for these data with coarser speckle illumination pattern to avoid speckle structure artefacts in the final image.

**Supplementary video 2: HiLo imaging of fixed and fluorescently stained mouse hippocampal neurons.** The first part of the video shows HiLo processed 25-image z-series (at 3 μm z-steps) of fixed and stained hippocampal mouse neurons obtained with the full FOV of the Mesolens. Raw data has been contrast adjusted for presentation. The second part of the movie is a zoom into a small region of interest (ROI) with only a few neurons in the FOV. The third part of the movie is a z-series of that ROI. The detail shown in the third part of the video is representative of the detail of the entire FOV without the need to re-acquire data. The zoom is purely digital. The specimen was excited at a wavelength of 488 nm and fluorescence was detected by the camera at an emission wavelength peak of 540 nm with 20 nm full-width-half-maximum spectral bandwidth. The sectioning parameter σ was set to 1 for these data. The apparent sweep of brightness across the specimen is a result of it not being flat relative to the direction of illumination.

**Supplementary video 3: Z-scan of the CLSM dataset of the same zebrafish as in supplementary video 1.** Excitation wavelength was 488 nm as before and fluorescence was detected at 525/39 nm bandpass. Images are Nyquist sampled at 3 px/µm lateral and 3 µm steps axial. Structure differs greatly from the HiLo processed data due to different illumination (epi-illumination for the CLSM data and transmission illumination for the HiLo data).

**Supplementary video 4: Eosin stained Daphnia larva.** Whole Daphnia larva excited at 488 nm and fluorescence detected at 540/20 nm as before. Dataset was subsampled to decrease video file size and the Fire LUT was chosen for contrast enhancement. Consequently, attenuated out-of-focus signal becomes more apparent and appears more significant than it is in terms of actual intensity. Optical sectioning parameter σ was set to 3 for this dataset.
